# Eosinophilic Esophagitis and Cow’s Milk: Mechanisms, Challenges, and Treatment Perspectives

**DOI:** 10.3390/nu17020265

**Published:** 2025-01-12

**Authors:** Giulio Dinardo, Alessandro Fiocchi, Maria Cristina Artesani, Paola De Angelis, Francesca Rea, Renato Tambucci, Lamia Dahdah, Vincenzo Fierro, Rocco Luigi Valluzzi, Stefania Arasi, Valentina Pecora, Arianna Cafarotti, Carmen Mazzuca, Cristiana Indolfi, Michele Miraglia del Giudice, Sara Urbani

**Affiliations:** 1 Department of Woman, Child and of General and Specialized Surgery, University of Campania Luigi Vanvitelli, 80138 Naples, Italy; cristianaind@hotmail.com (C.I.); michele.miragliadelgiudice@unicampania.it (M.M.d.G.); 2 Allergy Diseases Research Area, Pediatric Allergology Unit, Bambino Gesù Children’s Hospital, IRCCS, 00165 Rome, Italy; mariac.artesani@opbg.net (M.C.A.); lamiaantanios.dahdah@opbg.net (L.D.); vincenzo.fierro@opbg.net (V.F.); roccoluigi.valluzzi@opbg.net (R.L.V.); stefania.arasi@opbg.net (S.A.); valentina.pecora@opbg.net (V.P.); arianna.cafarotti@opbg.net (A.C.); carmen.mazzuca@opbg.net (C.M.); sara.urbani@opbg.net (S.U.); 3 Digestive Endoscopy Unit, Bambino Gesù Children’s Hospital, IRCCS, 00165 Rome, Italy; paola.deangelis@opbg.net (P.D.A.); francesca.rea@opbg.net (F.R.); renato.tambucci@opbg.net (R.T.)

**Keywords:** eosinophilic esophagitis, cow’s milk proteins, dietary therapy, elimination diet, milk allergy, esophageal inflammation, histologic remission, food hypersensitivity, Th2 immune response

## Abstract

Eosinophilic esophagitis is a chronic, antigen-driven, immune-mediated disease characterized by esophageal dysfunction and significant eosinophilic infiltration. Its rising incidence and prevalence over recent decades reflect both increased clinical awareness and the influence of environmental factors such as dietary patterns and allergen exposure. Among food allergens, cow’s milk proteins are the most commonly implicated triggers, contributing to esophageal inflammation through complex immunological pathways involving both IgE-mediated and non-IgE-mediated mechanisms. Dietary elimination of cow’s milk has been shown to induce histologic remission in over 60% of pediatric patients, underscoring its pivotal role in eosinophilic esophagitis management. Despite these promising results, challenges persist, including variability in individual responses, the burden of adherence to restrictive diets, and gaps in understanding the molecular mechanisms driving cow’s milk-induced esophageal inflammation. This review examines the complex relationship between eosinophilic esophagitis and cow’s milk, focusing on its role in disease pathogenesis and management, offering insights into its therapeutic implications. Understanding the interplay between eosinophilic esophagitis and dietary allergens, particularly cow’s milk, may inform the development of targeted interventions and improve clinical outcomes for affected patients.

## 1. Introduction

Eosinophilic esophagitis (EoE) is a chronic, progressive, antigen-mediated disease characterized by esophageal dysfunction symptoms and the presence of at least 15 eosinophils per high-power field (HPF) in the absence of other causes of esophageal eosinophilia [1]. EoE predominantly affects males, with a male-to-female ratio of 3:1, and shows a higher prevalence among Caucasians [2]. Symptoms vary with age: in infants and children, EoE often presents as feeding difficulties, failure to thrive, vomiting, or abdominal pain, while adolescents and adults typically experience persistent dysphagia and a high incidence of food bolus impaction [3]. The incidence and prevalence of EoE have risen significantly over the past few decades, now estimated at 5.31 per 100,000 individuals for incidence and 40.04 per 100,000 for prevalence [4]. EoE is diagnosed in approximately 6.5% of patients undergoing endoscopy for upper gastrointestinal symptoms, in 10% of those with non-obstructive dysphagia, and in up to 50% of patients with food bolus impaction [5]. Additionally, 1% of pediatric patients with gastroesophageal reflux disease (GERD) symptoms and 8–10% of those with GERD that is unresponsive to antacids are diagnosed with EoE [5]. Given the shared Th2-mediated pathogenic pathway, atopic comorbidities frequently occur alongside EoE [6]. There is an independent and cumulative association between atopic dermatitis, food allergy, allergic rhinitis, VKC, and asthma with the subsequent development of EoE [7,8,9]. A diagnosis of food allergy increases the risk of developing EoE ninefold [7], with cow’s milk (CM) allergy specifically raising the risk sixfold [10]. Notably, among patients with a history of anaphylaxis to CM, 38% demonstrate baseline esophageal eosinophilia (>15 eosinophils per HPF), even in the absence of chronic gastrointestinal symptoms [11]. Food antigens are recognized as key triggers of the inflammatory cascade in EoE, with CM emerging as one of the most implicated dietary allergens [12]. Clinical studies highlight that eliminating CM from the diet leads to significant symptom improvement and histologic remission, emphasizing its critical role in the disease’s pathogenesis. However, the precise mechanisms underlying cow’s milk-induced esophageal inflammation remain under investigation, involving both IgE-mediated and non-IgE-mediated immune responses. This review explores the interplay between EoE and cow’s milk, examining current evidence and evaluating its potential as a therapeutic target. By focusing on the role of cow’s milk in the development and management of EoE, we aim to identify novel strategies to improve patient outcomes.

## 2. Methods

This review was conducted as a narrative synthesis of the available literature to explore the relationship between eosinophilic esophagitis and cow’s milk. The literature search was performed in PubMed and Scopus databases. The studies considered for inclusion were those published in English from 2014 to 2024. Keywords employed in the search included “Eosinophilic Esophagitis”, “Cow’s Milk Proteins”, “Dietary Therapy”, “Elimination Diet”, “Milk Allergy”, and “Esophageal Inflammation”. Studies were selected based on their relevance to the topic, prioritizing clinical trials, systematic reviews, and meta-analyses.

## 3. The Potential Role of CM in EoE

### 3.1. Pathophysiological Insights

The underlying mechanisms by which cow’s milk proteins induce esophageal inflammation are not fully elucidated but are thought to involve both IgE-mediated and non-IgE-mediated pathways [1]. Proteins such as casein and whey have been implicated in the immune response, leading to the recruitment of eosinophils and the subsequent development of inflammation and esophageal remodeling [13]. Recent hypotheses suggest that the processing of milk significantly enhances its allergenic potential. Natural cow’s milk contains proteins like casein and whey, which serve as emulsifiers for fat globules. However, modern processing techniques, including ultra-heat treatment (UHT) and homogenization, fundamentally alter milk’s structure [14]. These processes reduce the fat globule size from micrometers to nanometers and lead to the denaturation of native proteins. The resulting lipid-protein nanoparticles, unique to processed milk, can penetrate the esophageal mucosa and act as immune adjuvants, stimulating elevated levels of IgG4 antibodies specific to proteins from cow’s milk, including α-lactalbumin (Bos d 4), β-lactoglobulin (Bos d 5), and casein (Bos d 8) [14,15]. An additional challenge with cow’s milk-induced esophageal inflammation is its role in oral immunotherapy (CM-OIT) for persistent food allergies [16,17,18]. While CM-OIT is effective in desensitizing patients to milk allergens, it carries a small but significant risk of triggering EoE. Retrospective and prospective studies report a prevalence of EoE ranging from 2% to 5.5% in patients undergoing CM-OIT, with symptoms emerging during both the escalation phase and the maintenance phase [16,19,20]. The incidence of this complication appears to be higher with cow’s milk compared to other foods [20]. In most cases, histological remission of EoE has been achieved only through strict avoidance of the triggering food and discontinuation of OIT [21].

### 3.2. Immunological Mechanisms Enhanced by Processed Milk

**Figure 1:**
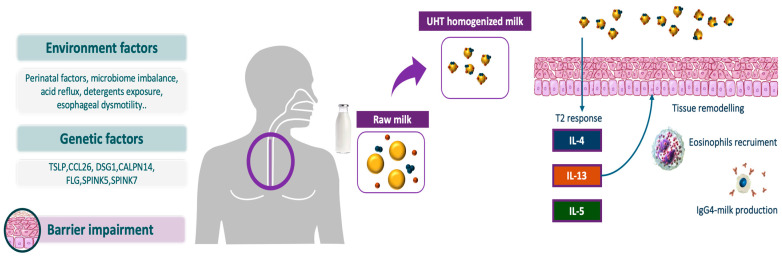
Environmental, genetic, and epithelial barrier impairment factors contribute to the pathogenesis of EoE [24,25,26,27,28,29,30,31,32]. Cow’s milk processing, such as UHT homogenization, alters the structure of milk proteins and lipids, leading to the formation of lipid-protein nanoparticles [14]. These nanoparticles can penetrate the esophageal mucosa, act as immune adjuvants, and drive a Th2 immune response characterized by the production of cytokines (IL-4, IL-5, IL-13), eosinophil recruitment, and subsequent tissue remodeling [13,14,15]. Additionally, these nanoparticles can stimulate an IgG4-mediated immune response. Although the precise role of IgG4 in EoE remains unclear, it may further amplify local inflammation, contributing to chronic antigen stimulation and inflammatory cell recruitment. This cascade creates a self-sustaining inflammatory loop characterized by persistent eosinophilic infiltration, epithelial damage, and the activation of fibroblasts and myofibroblasts. Ultimately, this process results in tissue remodeling, including subepithelial fibrosis and basal cell hyperplasia, leading to esophageal dysfunction.

Another aspect of cow’s milk proteins is that they can enhance the immune response by acting as antigens, capable of triggering an aberrant Th2 immune response in predisposed individuals [13,14]. This response is characterized by the production of cytokines such as interleukins (IL)-4, IL-5, and IL-13, which are critical in the recruitment and activation of eosinophils. IL-5 promotes eosinophil proliferation and survival, while IL-13 enhances epithelial barrier dysfunction and induces the expression of eotaxin-3, a chemokine that attracts eosinophils to the esophageal mucosa. This cascade creates a self-sustaining inflammatory loop, resulting in chronic eosinophilic infiltration and tissue remodeling [1,3]. The role of cow’s milk extends beyond Th2 responses. Non-IgE-mediated mechanisms, including delayed-type hypersensitivity reactions and the activation of other immune cells, such as mast cells and basophils, contribute to the pathogenesis [13,14]. These cells release mediators such as histamine and tryptase, exacerbating inflammation and contributing to symptoms like dysphagia and esophageal discomfort. Furthermore, cow’s milk proteins may impair epithelial integrity, facilitating antigen penetration and amplifying immune activation. Moreover, the denaturation of milk proteins, such as casein and β-lactoglobulin, during UHT reveals cryptic antigenic sites that are normally hidden in their native conformation [14]. These altered proteins adhere to the surface of lipid droplets, forming nanoparticles capable of crossing the esophageal mucosal barrier. This process could be exacerbated by conditions such as gastroesophageal reflux or the ingestion of synthetic surfactants used in milk stabilization [14]. Once in the mucosa, these nanoparticles interact with immune cells, driving a modified Th2 response characterized by the production of IL-4, IL-5, and IL-13. These cytokines recruit and activate eosinophils, initiating inflammation and esophageal remodeling. Furthermore, processed milk may also provoke an IgG4-dominated immune response, distinct from the IgE-mediated pathways seen in classic food allergies [14]. High levels of IgG4 antibodies specific to cow’s milk proteins have been detected in patients with EoE. These antibodies form immune complexes that accumulate in the esophageal wall, potentially amplifying local inflammation [15]. Recent preclinical studies provide additional insights into these mechanisms. Lozano-Ojalvo et al. identified distinct immune responses in EoE, where T follicular helper cells, producing IL-10 and IL-21, drive an IgG4-dominated pathway. This contrasts with milk allergy, where T follicular helper-13 cells dominate and promote IgE synthesis. Similarly, Cianferoni et al. demonstrated that peripheral blood mononuclear cells from EoE patients exhibit heightened TH2 activity, secreting IL-4, IL-5, and IL-13 in response to milk antigens. These findings underscore the critical role of T cell-mediated immunity and suggest that systemic and local immune activation against cow’s milk proteins are key contributors to EoE pathogenesis [22,23]. Together, these findings highlight the complex interplay of IgG4-dominated and non-IgE-mediated Th2 responses in driving cow’s milk-induced esophageal inflammation, paving the way for more targeted therapeutic approaches (Figure 1).

### 3.3. Genetic and Environmental Factors

Genetic predisposition plays a critical role in EoE. Most of the genetic loci implicated in EoE, as identified by genome-wide association studies (GWAS), are those involved in Th2-mediated immune responses and epithelial barrier functions [24]. Polymorphisms in the TSLP (thymic stromal lymphopoietin) gene, located on the 5q22 locus, have been strongly linked to EoE [25]. This gene, expressed in epithelial cells, encodes a cytokine involved in initiating type 2 inflammatory responses, including the recruitment of eosinophils and mast cells. Moreover, the gene encoding eotaxin-3 (CCL26) is overexpressed 53-fold in biopsy samples from patients with EoE compared to biopsies from healthy individuals [26]. Additionally, genes located in the epidermal differentiation complex (EDC) at 1q21 have been implicated in the pathogenesis of EoE [27]. Many of these genes are dysregulated in this condition, leading to epithelial barrier dysfunction and increased food antigen exposure. Among these, the filaggrin (FLG) and desmoglein-1 (DSG1) genes, which are critical for barrier function, are downregulated [28]. Conversely, calpain-14 (CAPN14), an esophagus-specific proteolytic enzyme induced by IL-13, is upregulated in EoE, resulting in further loss of DSG1 expression [29]. Finally, the serine peptidase inhibitors Kazal-type 5 and 7 (SPINK5 and SPINK7) contribute to the pathogenesis of EoE by modulating serine protease activity through downregulation [30]. Environmental factors further amplify the genetic susceptibility to EoE. Early life exposures, such as cesarean delivery, formula feeding, and antibiotic use, may disrupt microbial diversity, reducing immune tolerance and predisposing individuals to food-triggered inflammation. The composition of the esophageal microbiome, including imbalances in bacteria like Firmicutes and Bacteroides, has been identified as a contributing factor. Alterations in microbial communities could exacerbate esophageal inflammation, especially in genetically predisposed individuals. Polymorphisms in genes regulating epithelial integrity can compromise the esophageal lining, allowing allergens to penetrate and trigger immune responses. This barrier dysfunction is further aggravated by environmental insults, such as acid reflux, detergent exposure, or infection, reinforcing the need for targeted therapies that address both immune dysregulation and epithelial repair [31,32].

### 3.4. Long-Term Structural and Functional Impact on the Esophagus

Persistent exposure to cow’s milk proteins in EoE can lead to significant structural changes in the esophagus [33]. Chronic eosinophilic inflammation results in epithelial hyperplasia, subepithelial fibrosis, and the formation of strictures [34]. These changes are driven by the secretion of growth factors, such as TGF-β (transforming growth factor-beta), which promote fibroblast activation and extracellular matrix deposition. The narrowing and stiffening of the esophageal lumen contribute to the hallmark clinical manifestations of EoE, including dysphagia and food impaction [34]. Importantly, these changes, as highlighted by some studies, appear to be reversible with the removal of cow’s milk from the diet, emphasizing its critical role in disease progression.

## 4. From Elemental Diet to CM Elimination Diet: Evolving Dietary Strategies

**Figure 2:**
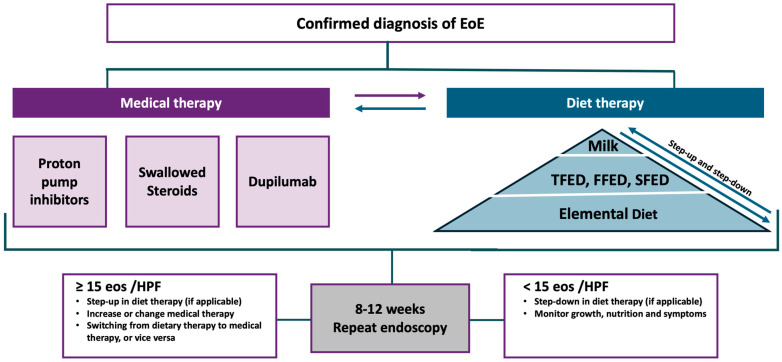
Algorithm for the management of EoE. Following a confirmed diagnosis, treatment options include medical therapy—proton pump inhibitors, swallowed steroids, and dupilumab—and dietary therapy, ranging from milk exclusion to empirical elimination diets (TFED, FFED, SFED) and elemental diets. The approach is guided by patient response, assessed after 8–12 weeks via repeat endoscopy and histological evaluation [35,36]. TFED: two-food elimination diet; FFED: four-food elimination diet; SFED: six-food elimination diet; eos: eosinophils; HPF: high-power field.

Current guidelines incorporate the use of pharmacological therapies—specifically proton pump inhibitors and swallowed corticosteroids—as first-line strategies alongside dietary interventions in the therapeutic management of the condition [35] (Figure 2).

**Figure 3:**
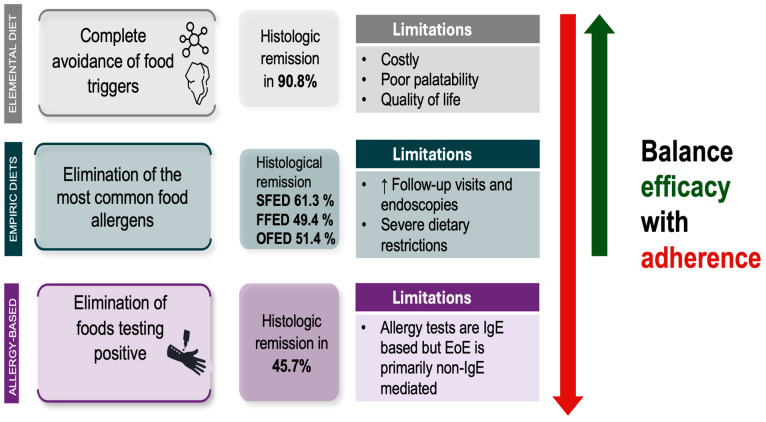
Comparison of dietary strategies for the management of eosinophilic esophagitis (EoE), highlighting efficacy and limitations [12].

Three main dietary approaches have been explored: exclusive elemental diets, allergy test-guided elimination diets, and empiric elimination diets (Figure 3). Each approach aims to minimize exposure to dietary allergens and identify specific trigger foods through reintroduction protocols. These strategies vary in terms of efficacy, practicality, and their impact on quality of life, with outcomes often differing between children and adults. Notably, dietary therapies tend to be more effective in pediatric populations, particularly in cases of very early-onset EoE, which show a significant response to elimination diets [37,38]. The efficacy of each dietary strategy must be carefully balanced against its limitations, as these can significantly impact patient adherence [39].

### 4.1. Exclusive Elemental Diets: High Efficacy, Limited Practicality

Elemental diets, which use amino acid-based formulas devoid of antigenic capacity, remain the most effective dietary intervention for EoE. The first evidence of their efficacy in a pediatric population was provided by Kelly et al. in 1995 [40]. While elemental diets achieve histologic remission in 91% of patients, with a dramatic reduction in esophageal eosinophilia and symptom resolution, they are associated with significant limitations [41]. Poor palatability often necessitates the use of nasogastric feeding tubes, and their high cost, along with negative impacts on social and psychological well-being, makes them challenging to sustain [42]. Additionally, caution is required in children under 2 years of age. Long-term avoidance of solid foods in these patients can lead to delayed development of oral-motor skills. Consequently, elemental diets are now reserved for specific cases, such as refractory EoE, or are employed as a bridge therapy while awaiting alternative treatments [43].

### 4.2. Allergy Based-Test Elimination Diets: Modest Success and Limited Reliability

Targeted elimination diets (TEDs), guided by food allergy tests such as skin prick tests (SPT), patch tests, and serum-specific IgE measurements, have been explored as a treatment strategy for EoE [44,45,46]. However, their overall effectiveness has proven limited, with histologic remission achieved in only 45.7% of patients, as confirmed by a recent meta-analysis [12]. This modest success can be attributed to the predominantly non-IgE–mediated nature of EoE, which undermines the predictive accuracy of IgE-based tests in identifying trigger foods. The heterogeneity across studies, stemming from variations in allergy testing techniques, allergens assessed, and outcome definitions, further weakens their clinical reliability. Consequently, clinical guidelines no longer recommend allergy testing as a primary method for identifying dietary triggers in EoE [47].

### 4.3. Empiric Elimination Diets: A Practical, Effective, and Adaptable Approach

Empiric elimination diets have proven effective in the management of EoE, with the six-food elimination diet (SFED) being the first studied approach. Kagalwalla et al. demonstrated that excluding six common allergenic food groups—milk, soy, egg, wheat, peanuts/tree nuts, and seafood—achieved histologic remission in 74% of pediatric patients after 6 weeks. Sequential reintroduction of foods identified cow’s milk as the most common trigger (74%), followed by wheat (26%) and egg (17%) [48,49]. However, SFED requires significant dietary restrictions and multiple endoscopies, making it challenging to implement and impacting quality of life. To address these limitations, simplified approaches such as the four-food elimination diet (FFED), two-food elimination diet (targeting milk and wheat) and one-food elimination diet (OFED) (excluding milk) have been proposed [50,51,52]. The efficacy of these different approaches was discussed by Mayerhofer et al. in a comprehensive meta-analysis that compared SFED, FFED, OFED, and TED [12]. It included data from 34 studies encompassing 1762 patients, 915 of whom were children. The overall histologic remission rate for all dietary approaches was 53.8% [12]. Among individual diets, SFED had the highest remission rate at 61.3%, followed by OFED (51.4%), FFED (49.4%), and TED (45.7%). These findings suggest that while SFED remains the most effective regimen, the differences in remission rates across the diets were not statistically significant, as confirmed by meta-regression analysis. Clinical response rates were generally higher than histologic remission rates, with an overall response rate of 80.8%. SFED again led with a clinical response rate of 92.8%, followed by OFED (87.1%), FFED (74.1%), and TED (69.0%). Interestingly, the correlation between histologic and clinical responses was weak, underscoring the complex relationship between symptom relief and histologic changes. The meta-analysis revealed comparable remission rates for pediatric and adult populations. Pediatric patients had a histologic remission rate of 57.2%, slightly higher than adults at 48.9%. In pediatric subgroups, OFED performed particularly well, suggesting that it may be a suitable alternative to more restrictive diets like SFED in younger patients. The findings highlight the potential for less restrictive diets, such as OFED and FFED, to serve as primary treatment options for EoE. While SFED is the most effective, its complexity and impact on quality of life can hinder adherence, particularly in long-term maintenance. OFED, which focuses on eliminating only cow’s milk—a common trigger in EoE—offers a balance between efficacy and practicality [12]. A recent randomized study compared OFED, excluding milk, to FFED, which excludes milk, egg, wheat, and soy, in children with EoE [53]. Over 12 weeks, both diets showed comparable histologic remission rates (44% for OFED and 41% for FFED, *p* = 1.00) with minimal differences in complete remission rate (<1 eos/hpf). Parent-reported symptoms improved more with FFED than OFED (−25.0 vs. −14.5 in PEESS scores, *p* = 0.04), particularly for reflux and nausea. However, child-reported symptom outcomes and endoscopic findings were similar between the two groups. Baseline milk-specific IgG4 levels were higher in OFED responders, suggesting potential as a predictive biomarker. Adherence was higher in OFED (10.5% dropout vs. 32% for FFED), reflecting the greater complexity and social burden of multi-food elimination. While FFED provided slightly better symptom relief, OFED’s simplicity and tolerability make it a practical first-line option, especially since milk is the most common trigger in EoE. Both diets highlighted the dissociation between symptom improvement and histologic remission, emphasizing the need for tailored and comprehensive treatment strategies [53].

## 5. Evidence Supporting CM Elimination Diet

The elimination of cow’s milk as a single dietary intervention has been shown to induce significant clinical and histologic improvement in a substantial proportion of children with EoE. Studies report that strict elimination, which removes all sources of milk—including baked goods and trace amounts—is associated with histologic remission rates of approximately 67%. Liberalized elimination diets, which permit traces of milk in processed or baked forms, demonstrate lower remission rates (29%) but may offer greater dietary flexibility and potentially reduce the risk of developing new IgE-mediated allergies [33].

In one of the first studies achieved by Kagalwalla et al., 17 children with an average age of 5.5 years were treated with Cow’s Milk Elimination (CME) for at least six weeks [52]. The cohort, predominantly male (70.6%) and white (82.4%), also included a high proportion (88%) of atopic individuals. Histologic remission, defined as <15 eosinophils per high-power field (eos/hpf), was achieved in 64.7% of participants, with 23.6% reaching complete remission (<1 eos/hpf). Importantly, even among non-remitters, a 33% reduction in eosinophil counts was observed, and all participants experienced resolution of clinical symptoms such as dysphagia and abdominal pain. When compared to other interventions, such as SFED (63% remission) and topical steroids (62% remission), CME demonstrated similar effectiveness but was less efficacious than elemental diets, which achieved an 83% remission rate [52].

A subsequent investigation conducted a larger retrospective study comprising 152 children [54]. Of these, 102 followed a dairy-free diet (DFD), and 50 adhered to SFED. The mean age was higher than in Kagalwalla’s study (9.2 years vs. 5.5 years), but similar demographic trends were observed, with a predominantly male (76.3%), white (69.3%), and atopic (90.8%) population [52,54]. Among DFD participants, 56.9% achieved histologic remission (<15 eos/hpf), which was comparable to the SFED group. However, the mean post-treatment eosinophil count in the DFD group remained above the remission threshold (20.6 eos/hpf), suggesting incomplete histologic improvement in some cases. Both groups demonstrated significant eosinophil reductions regardless of treatment duration (<10, 10–12, or >12 weeks), with no significant differences in efficacy across time points. Notably, children receiving both DFD and concurrent proton pump inhibitor therapy showed higher remission rates, indicating a synergistic effect between diet and pharmacological management [54].

A prospective study by Kruszewski et al. evaluated CME against swallowed fluticasone therapy in a cohort of 44 children newly diagnosed with EoE [55]. The mean age of participants was 12–13 years, slightly older than in previous studies, and 63–65% were male. Over a treatment period of 6–8 weeks, CME resulted in histologic remission in 64% of participants, while fluticasone was more effective, achieving remission in 80%. Despite this, CME still demonstrated significant efficacy, with a mean reduction in eosinophil counts of 29 eos/hpf (*p* = 0.006), bringing patients close to the remission threshold. Moreover, CME was associated with greater improvements in quality of life (QoL) scores compared to fluticasone. However, the high attrition rate in the CME group (30%) highlighted challenges with adherence, potentially limiting its generalizability [33,55].

Wechsler et al. conducted a more recent prospective study in 2022, focusing on CME’s histologic, symptomatic, and QoL outcomes [56]. The study enrolled 54 children, of whom 41 completed the intervention. The mean age was 9 years, younger than in Kruszewski’s study but comparable to Wong’s cohort [54,55]. Histologic remission (<15 eos/hpf) was achieved in 51% of participants, with a significant reduction in eosinophil counts among non-remitters (*p* < 0.001). Additionally, 29% of children reported complete symptom resolution, and a trend toward improved QoL was observed [56].

A study involving 31 children evaluated the outcomes of exclusive CME over a median duration of three months [13]. Participants had a median age of 9 years, with 84% male and 74% Caucasian. A significant proportion had atopic conditions such as eczema (57%) and asthma (50%). Histologic remission (<15 eos/hpf) was achieved in 58% of patients, while 23% reached complete remission (<1 eos/hpf). Symptom improvement was notable, with 90% reporting relief, including significant reductions in dysphagia, vomiting, and abdominal pain. When comparing strict CME to a liberalized approach allowing trace amounts of milk, remission rates were higher in the strict group (67% vs. 29%), though the difference was not statistically significant. This highlights the importance of adherence and the potential limitations of partial elimination diets in achieving optimal outcomes. Notably, 77% of non-remitters experienced some reduction in eosinophil counts, suggesting that CME provides symptom relief even when complete histologic remission is not achieved. CME’s remission rates are slightly lower than those observed with more extensive elimination strategies, such as the six-food elimination diet (73.1%) or elemental diets (83%), but comparable to pharmacologic interventions like topical corticosteroids. While CME offers a targeted approach, its effectiveness appears to be limited in cases where multiple food triggers are involved. Moreover, non-remitters may require additional interventions, such as broader dietary elimination or corticosteroid therapy [13].

All of these studies collectively demonstrate the potential of CME to achieve significant clinical and histologic improvements in children with EoE (Table 1). While its remission rates may be lower than those of elemental diets or pharmacologic options like fluticasone, CME offers a non-invasive, dietary-based alternative with fewer side effects [33]. In addition to achieving histologic remission, the removal of cow’s milk from the diet significantly improves clinical symptoms such as dysphagia, vomiting, and abdominal pain. Symptom improvement has been reported in up to 90% of patients, regardless of histologic outcome. Notably, cow’s milk elimination appears to outperform other single-food elimination strategies. For example, milk is eight times more likely than wheat to induce EoE recurrence upon reintroduction, further highlighting its pivotal role in disease pathogenesis. Furthermore, elimination diets that target cow’s milk alone are often preferred over more restrictive multi-food elimination diets due to their lower impact on nutritional status, quality of life, and the need for invasive follow-up procedures. Factors such as patient adherence, concurrent therapies, and the quality of dietary education significantly influence outcomes, underscoring the need for individualized approaches to maximize its efficacy.

## 6. Discussion

The comparison between single-food elimination diets, specifically CME, and multi-food elimination diets, such as FFED or SFED, reveals important insights into their efficacy and impact on patient quality of life. While multi-food elimination diets often show slightly higher remission rates, the differences are not always statistically significant, and single-food elimination diets, particularly those targeting cow’s milk, offer comparable outcomes with significantly fewer dietary restrictions [12,53,56].

### 6.1. Efficacy Comparison

Evidence consistently demonstrates that cow’s milk is the most common trigger in EoE, making CME an effective and pragmatic first-line approach. For instance, Kagalwalla et al. reported a 64.7% histologic remission rate for CME, comparable to SFED (63%) and pharmacologic interventions like topical corticosteroids (62%), but lower than elemental diets (83%). This finding is echoed in studies such as those by Wong et al. and Teoh et al., who found remission rates for CME ranging between 51% and 67%, depending on the strictness of the elimination protocol [13,54]. By contrast, multi-food elimination diets, such as FFED and SFED, have shown remission rates of 41% to 61%, respectively. The meta-analysis by Mayerhofer et al. revealed that while SFED achieved the highest remission rate at 61.3%, single-food elimination diets like CME performed similarly, with a remission rate of 51.4%. This aligns with findings from Kliewer et al., who observed that CME and FFED resulted in similar histologic remission rates (44% vs. 41%) and minimal differences in complete remission (<1 eos/hpf). These results suggest that eliminating additional foods may not always yield a significant increase in efficacy compared to targeting the primary allergen [12].

### 6.2. Quality of Life and Adherence

While multi-food elimination diets may marginally outperform CME in some cases, their complexity poses challenges to long-term adherence and patient satisfaction. Restrictive diets, such as SFED, often disrupt daily routines, social interactions, and nutritional balance, particularly in children. Mayerhofer et al. highlighted that SFED’s complexity significantly impacts quality of life, leading to higher dropout rates. Similarly, Kliewer et al. found that adherence was substantially higher for CME (10.5% dropout) compared to FFED (32%) [12]. The simplicity of CME reduces dietary restrictions, minimizing the psychological burden on patients and their families. Another advantage of CME is its ability to maintain efficacy while being more practical for patients. For example, Teoh et al. demonstrated that even non-remitters on CME experienced significant symptom improvement (77%), with 90% reporting relief from dysphagia, vomiting, and abdominal pain. This symptom relief, regardless of histologic remission, underscores CME’s value as a therapeutic option that balances effectiveness with patient comfort [12,33].

### 6.3. Challenges and Future Directions

Despite its demonstrated efficacy, several challenges remain with the use of cow’s milk elimination diets. Most patients with EoE triggered by dairy proteins can tolerate extensively hydrolyzed formulas, which have significantly reduced allergenic potential. These formulas are currently recommended as a first-line dietary intervention for infants with EoE, as they provide adequate nutritional support [57]. However, these formulas are expensive and not very palatable, which can make it hard for patients to follow the treatment. Adherence to strict elimination can be difficult, particularly in children. Studies suggest that reintroducing baked milk gradually may help to maintain oral tolerance and reduce the risk of recurrence [58]. A prospective study found that 75% of patients with milk-induced EoE tolerated milk heated above 100 °C for 20 min without triggering inflammation or symptoms [59]. Dietary management for EoE should be personalized based on disease severity, dietary preferences, and known triggers. CME is often the first step, offering significant symptom relief with fewer restrictions compared to multi-food elimination diets like SFED, which are used for patients who do not respond to CME or have multiple triggers. A step-up approach, starting with less restrictive diets and progressing as needed, can reduce the burden on patients and families while improving adherence [13]. EoE triggered by cow’s milk may be linked to structural changes caused by milk processing, but the exact role of milk proteins in the disease is not yet understood. Further research is needed to explore how these proteins interact with the immune system to drive EoE pathogenesis. Additionally, integrating predictive biomarkers, such as the IgG4/IgE ratio observed in some responders to single-food elimination diets, may enhance the ability to personalize dietary interventions [55]. By personalizing dietary strategies and using a step-up approach, clinicians can balance effective treatment with patients’ quality of life. This approach could streamline dietary management, reduce the need for broader eliminations, and optimize patient outcomes. Advancing diagnostic tools and personalized dietary strategies will further refine this patient-centered approach, ensuring an optimal balance between efficacy and quality of life in EoE management.

### 6.4. Study Limitations

The heterogeneity among the studies reviewed, including differences in methodologies, populations, and outcome measures, poses challenges to the generalizability of the findings. Practical issues, such as adherence to dietary interventions and their potential nutritional impacts, may limit the real-world applicability of the proposed strategies. Additionally, despite progress in understanding the mechanisms underlying cow’s milk-induced EoE, significant gaps remain, particularly regarding the role of processed milk in enhancing allergenicity. Many of the reviewed studies rely on small sample sizes or retrospective data, which can introduce bias and affect the robustness of conclusions. Moreover, the lack of long-term data prevents a comprehensive evaluation of the sustainability of remission and the broader impacts on patients’ quality of life. These limitations highlight the need for further research to refine therapeutic approaches and improve outcomes for individuals with EoE.

## 7. Conclusions

CME represents a viable first-line therapy for EoE, particularly for families seeking dietary rather than pharmacologic interventions. Its moderate effectiveness highlights the need for further research to identify predictors of response and optimize its use in clinical practice. A critical balance between dietary adherence, remission goals, and quality of life must guide its application. In conclusion, cow’s milk plays a central role in the pathogenesis and management of EoE. Its removal has emerged as a cornerstone of dietary therapy, offering a targeted approach to achieve remission and symptom control in affected children. However, individualized treatment plans, informed by ongoing research, are essential to balance efficacy, adherence, and quality of life.

## Figures and Tables

**Table 1:** Comparison of dietary elimination strategies in eosinophilic esophagitis.

Study	Diet Type	Participants	Histologic Remission Rates *	Symptom Improvement **	Key Findings
Kagalwalla et al. [52]	CME (Strict)	17 children (mean age 5.5 years)	64.7% (<15 eos/hpf), 23.6% (<1 eos/hpf)	100% resolution of symptoms	Similar efficacy to SFED (63%) and topical steroids (62%), less effective than elemental diets (83%).
Wong et al. [54]	CME (Strict vs. Liberalized)	152 children (mean age 9.2 years)	56.9% for CME, comparable to SFED	Significant symptom reduction; PPI adjunct increased remission	Strict CME more effective than liberalized CME (67% vs. 29%), but both provided symptom relief.
Kruszewski et al. [55]	CME vs. Fluticasone	44 children (mean age 12–13 years)	64% for CME, 80% for fluticasone	Greater QoL improvement with CME	CME demonstrated significant eosinophil reduction (*p* = 0.006) and strong QoL outcomes.
Wechsler et al. [56]	CME (Strict)	54 children (mean age 9 years)	51% histologic remission	29% complete symptom resolution	Intensive dietary education enhanced adherence; significant eosinophil reduction even in non-remitters.
Teoh et al. [13]	CME (Strict vs. Liberalized)	31 children (median age 9 years)	58% (strict CME), 67% for strict subgroup	90% symptom improvement	Liberalized CME showed lower remission (29%), highlighting the need for strict adherence in most cases.
Kliewer et al. [53]	OFED vs. FFED	63 children (ages 6–17 years)	44% (OFED), 41% (FFED)	Higher parent-reported improvement for FFED	Adherence was better for OFED (10.5% dropout vs. 32% for FFED); simplicity of CME supports first-line use.
Mayerhofer et al. [12]	OFED, FFED, SFED, TED	Meta-analysis (1762 patients)	51.4% (OFED), 49.4% (FFED), 61.3% (SFED)	80.8% overall clinical response	SFED most effective for histologic remission; OFED effective with fewer dietary restrictions.

* Histologic Remission: Reduction in eosinophil counts (<15 eos/hpf) after treatment. ** Symptom Improvement: Relief in symptoms like dysphagia, vomiting, or abdominal pain, often subjectively assessed.

## References

[1] de Bortoli N., Visaggi P., Penagini R., Annibale B., Baiano Svizzero F., Barbara G., Bartolo O., Battaglia E., Di Sabatino A., De Angelis P. (2024). The 1st EoETALY Consensus on the Diagnosis and Management of Eosinophilic Esophagitis—Definition, Clinical Presentation and Diagnosis. Dig. Liver Dis..

[2] Dellon E.S. (2014). Epidemiology of Eosinophilic Esophagitis. Gastroenterol. Clin. N. Am..

[3] Visaggi P., Savarino E., Sciume G., Di Chio T., Bronzini F., Tolone S., Frazzoni M., Pugno C., Ghisa M., Bertani L. (2021). Eosinophilic Esophagitis: Clinical, Endoscopic, Histologic and Therapeutic Differences and Similarities between Children and Adults. Therap. Adv. Gastroenterol..

[4] Hahn J.W., Lee K., Shin J.I., Cho S.H., Turner S., Shin J.U., Yeniova A.Ö., Koyanagi A., Jacob L., Smith L. (2023). Global Incidence and Prevalence of Eosinophilic Esophagitis, 1976-2022: A Systematic Review and Meta-Analysis. Clin. Gastroenterol. Hepatol..

[5] Soon I.S., Butzner J.D., Kaplan G.G., Debruyn J.C.C. (2013). Incidence and Prevalence of Eosinophilic Esophagitis in Children. J. Pediatr. Gastroenterol. Nutr..

[6] Capucilli P., Hill D.A. (2019). Allergic Comorbidity in Eosinophilic Esophagitis: Mechanistic Relevance and Clinical Implications. Clin. Rev. Allergy Immunol..

[7] Hill D.A., Grundmeier R.W., Ramos M., Spergel J.M. (2018). Eosinophilic Esophagitis Is a Late Manifestation of the Allergic March. J. Allergy Clin. Immunol. Pract..

[8] Artesani M.C., Urbani S., Riccardi C., Esposito M., De Angelis P., Rea F., Tambucci R., Malamisura M., Buzzonetti L., Fiocchi A.G. (2024). Vernal Keratoconjunctivitis and Eosinophilic Esophagitis: A Rare Combination?. World Allergy Organ. J..

[9] Ridolo E., Nicoletta F., Lombardi C., Passalacqua G., Senna G., Canonica G.W. (2024). Eosinophilic Esophagitis and Inhalant Antigens: Pointing out the Roles of Allergic Rhinitis, Immunotherapy and Biologic Treatment. World Allergy Organ. J..

[10] Haber C., Al-Shaikhly T., Jhaveri P. (2024). Milk or Egg Allergy Diagnosis Increases the Risk of Eosinophilic Esophagitis Diagnosis. J. Allergy Clin. Immunol. Pract..

[11] Barbosa A.C., Castro F.M., Meireles P.R., Arruda L.K., Cardoso S.R., Kalil J., Yang A.C. (2018). Eosinophilic Esophagitis: Latent Disease in Patients with Anaphylactic Reaction to Cow’s Milk. J. Allergy Clin. Immunol. Pract..

[12] Mayerhofer C., Kavallar A.M., Aldrian D., Lindner A.K., Müller T., Vogel G.F. (2023). Efficacy of Elimination Diets in Eosinophilic Esophagitis: A Systematic Review and Meta-Analysis. Clin. Gastroenterol. Hepatol..

[13] Teoh T., Mill C., Chan E., Zimmer P., Avinashi V. (2019). Liberalized Versus Strict Cow’s Milk Elimination for the Treatment of Children with Eosinophilic Esophagitis. J. Can. Assoc. Gastroenterol..

[14] Baker J.R., Gangwar R.S., Platts-Mills T.A. (2024). The Processed Milk Hypothesis: A Major Factor in the Development of Eosinophilic Esophagitis (EoE)?. J. Allergy Clin. Immunol..

[15] Schuyler A.J., Wilson J.M., Tripathi A., Commins S.P., Ogbogu P.U., Kruzsewski P.G., Barnes B.H., McGowan E.C., Workman L.J., Lidholm J. (2018). Specific IgG4 Antibodies to Cow’s Milk Proteins in Pediatric Patients with Eosinophilic Esophagitis. J. Allergy Clin. Immunol..

[16] Sánchez-García S., Rodríguez Del Río P., Escudero C., Martínez-Gómez M.J., Ibáñez M.D. (2012). Possible Eosinophilic Esophagitis Induced by Milk Oral Immunotherapy. J. Allergy Clin. Immunol..

[17] Bognanni A., Chu D.K., Firmino R.T., Arasi S., Waffenschmidt S., Agarwal A., Dziechciarz P., Horvath A., Jebai R., Mihara H. (2022). World Allergy Organization (WAO) Diagnosis and Rationale for Action against Cow’s Milk Allergy (DRACMA) Guideline Update—XIII—Oral Immunotherapy for CMA—Systematic Review. World Allergy Organ. J..

[18] Brozek J.L., Firmino R.T., Bognanni A., Arasi S., Ansotegui I., Assa’ad A.H., Bahna S.L., Canani R.B., Bozzola M., Chu D.K. (2022). World Allergy Organization (WAO) Diagnosis and Rationale for Action against Cow’s Milk Allergy (DRACMA) Guideline Update—XIV—Recommendations on CMA Immunotherapy. World Allergy Organ. J..

[19] Winter C., Babaie D., Nabavi M., Arshi S., Mesdaghi M., Chavoshzadeh Z., Hasan Bemanian M., Tafakori M., Amirmoini M., Esmailzadeh H. (2017). Cow’ s Milk Desensitization in Anaphylactic Patients: A New Personalized-Dose Method. Iran. J. Allergy Asthma Immunol..

[20] Morales-Cabeza C., Infante S., Cabrera-Freitag P., Fuentes-Aparicio V., Zubeldia J.M., Álvarez-Perea A. (2023). Oral Immunotherapy and Risk of Eosinophilic Esophagitis in Children: 15 Years’ Experience. J. Pediatr. Gastroenterol. Nutr..

[21] Babaie D., Mesdaghi M., Nishino M., Mansouri M., Ebisawa M. (2017). Oral and Sublingual Immunotherapy: Potential Causes for Eosinophilic Gastrointestinal Disorders?. Int. Arch. Allergy Immunol..

[22] Lozano-Ojalvo D., Chen X., Kazmi W., Menchén-Martínez D., Pérez-Rodríguez L., Fernandes-Braga W., Tyler S., Benkov K., Pittman N., Lai J. (2024). Differential T Follicular Helper Cell Phenotypes Distinguish IgE-Mediated Milk Allergy from Eosinophilic Esophagitis in Children. J. Allergy Clin. Immunol..

[23] Cianferoni A., Ruffner M.A., Guzek R., Guan S., Brown-Whitehorn T., Muir A., Spergel J.M. (2018). Elevated Expression of Activated TH2 Cells and Milk-Specific TH2 Cells in Milk-Induced Eosinophilic Esophagitis. Ann. Allergy Asthma Immunol..

[24] Chang X., March M., Mentch F., Nguyen K., Glessner J., Qu H., Liu Y., Furuta G., Aceves S., Gonsalves N. (2022). A Genome-Wide Association Meta-Analysis Identifies New Eosinophilic Esophagitis Loci. J. Allergy Clin. Immunol..

[25] Sherrill J.D., Gao P.S., Stucke E.M., Blanchard C., Collins M.H., Putnam P.E., Franciosi J.P., Kushner J.P., Abonia J.P., Assa’ad A.H. (2010). Variants of Thymic Stromal Lymphopoietin and Its Receptor Associate with Eosinophilic Esophagitis. J. Allergy Clin. Immunol..

[26] Blanchard C., Wang N., Stringer K.F., Mishra A., Fulkerson P.C., Abonia J.P., Jameson S.C., Kirby C., Konikoff M.R., Collins M.H. (2006). Eotaxin-3 and a Uniquely Conserved Gene-Expression Profile in Eosinophilic Esophagitis. J. Clin. Investig..

[27] Blanchard C., Stucke E.M., Burwinkel K., Caldwell J.M., Collins M.H., Ahrens A., Buckmeier B.K., Jameson S.C., Greenberg A., Kaul A. (2010). Coordinate Interaction between IL-13 and Epithelial Differentiation Cluster Genes in Eosinophilic Esophagitis. J. Immunol..

[28] Kottyan L.C., Rothenberg M.E. (2017). Genetics of Eosinophilic Esophagitis. Mucosal Immunol..

[29] Litosh V.A., Rochman M., Rymer J.K., Porollo A., Kottyan L.C., Rothenberg M.E. (2017). Calpain-14 and Its Association with Eosinophilic Esophagitis. J. Allergy Clin. Immunol..

[30] Azouz N.P., Ynga-Durand M.A., Caldwell J.M., Jain A., Rochman M., Fischesser D.M., Ray L.M., Bedard M.C., Mingler M.K., Forney C. (2018). The Antiprotease SPINK7 Serves as an Inhibitory Checkpoint for Esophageal Epithelial Inflammatory Responses. Sci. Transl. Med..

[31] Khan S., Guo X., Liu T., Iqbal M., Jiang K., Zhu L., Chen X., Wang B.M. (2021). An Update on Eosinophilic Esophagitis: Etiological Factors, Coexisting Diseases, and Complications. Digestion.

[32] Torrijos E.G., Gonzalez-Mendiola R., Alvarado M., Avila R., Prieto-Garcia A., Valbuena T., Borja J., Infante S., Lopez M.P., Marchan E. (2018). Eosinophilic Esophagitis: Review and Update. Front. Med..

[33] Grasso J., Radler D.R., Zelig R. (2024). Single-Food Elimination of Cow’s Milk as a Treatment for Eosinophilic Esophagitis in Children Aged 2-18 Years: A Review of the Literature. Nutr. Clin. Pract..

[34] Shaker A. (2024). Esophageal Remodeling in Eosinophilic Esophagitis. Curr. Opin. Gastroenterol..

[35] Amil-Dias J., Oliva S., Papadopoulou A., Thomson M., Gutiérrez-Junquera C., Kalach N., Orel R., Auth M.K.H., Nijenhuis-Hendriks D., Strisciuglio C. (2024). Diagnosis and Management of Eosinophilic Esophagitis in Children: An Update from the European Society for Paediatric Gastroenterology, Hepatology and Nutrition (ESPGHAN). J. Pediatr. Gastroenterol. Nutr..

[36] Savarino E.V., Barbara G., Bilò M.B., De Bortoli N., Di Sabatino A., Oliva S., Penagini R., Racca F., Tortora A., Rumi F. (2024). Eosinophilic Esophagitis in Adults and Adolescents: Epidemiology, Diagnostic Challenges, and Management Strategies for a Type 2 Inflammatory Disease. Ther. Adv. Gastroenterol..

[37] Arias Á., Tejera-Muñoz A., Gutiérrez-Ramírez L., Molina-Infante J., Lucendo A.J. (2024). Efficacy of Dietary Therapy for Eosinophilic Esophagitis in Children and Adults: An Updated Systematic Review and Meta-Analysis. Nutrients.

[38] Lyles J.L., Martin L.J., Shoda T., Collins M.H., Trimarchi M.P., He H., Kottyan L.C., Mukkada V.A., Rothenberg M.E. (2021). Very Early Onset Eosinophilic Esophagitis Is Common, Responds to Standard Therapy, and Demonstrates Enrichment for CAPN14 Genetic Variants. J. Allergy Clin. Immunol..

[39] Votto M., De Filippo M., Lenti M.V., Rossi C.M., Di Sabatino A., Marseglia G.L., Licari A. (2022). Diet Therapy in Eosinophilic Esophagitis. Focus on a Personalized Approach. Front. Pediatr..

[40] Kelly K.J., Lazenby A.J., Rowe P.C., Yardley J.H., Perman J.A., Sampson H.A. (1995). Eosinophilic Esophagitis Attributed to Gastroesophageal Reflux: Improvement with an Amino Acid-Based Formula. Gastroenterology.

[41] Arias Á., González-Cervera J., Tenias J.M., Lucendo A.J. (2014). Efficacy of Dietary Interventions for Inducing Histologic Remission in Patients with Eosinophilic Esophagitis: A Systematic Review and Meta-Analysis. Gastroenterology.

[42] Molina-Infante J., Lucendo A.J. (2018). Dietary Therapy for Eosinophilic Esophagitis. J. Allergy Clin. Immunol..

[43] Delaney A.L., Arvedson J.C. (2008). Development of Swallowing and Feeding: Prenatal through First Year of Life. Dev. Disabil. Res. Rev..

[44] Spergel J.M., Beausoleil J.L., Mascarenhas M., Liacouras C.A. (2002). The Use of Skin Prick Tests and Patch Tests to Identify Causative Foods in Eosinophilic Esophagitis. J. Allergy Clin. Immunol..

[45] Spergel J.M., Brown-Whitehorn T.F., Cianferoni A., Shuker M., Wang M.L., Verma R., Liacouras C.A. (2012). Identification of Causative Foods in Children with Eosinophilic Esophagitis Treated with an Elimination Diet. J. Allergy Clin. Immunol..

[46] Philpott H., Nandurkar S., Royce S.G., Thien F., Gibson P.R. (2016). Allergy Tests Do Not Predict Food Triggers in Adult Patients with Eosinophilic Oesophagitis. A Comprehensive Prospective Study Using Five Modalities. Aliment. Pharmacol. Ther..

[47] Dhar A., Haboubi H.N., Attwood S.E., Auth M.K.H., Dunn J.M., Sweis R., Morris D., Epstein J., Novelli M.R., Hunter H. (2022). British Society of Gastroenterology (BSG) and British Society of Paediatric Gastroenterology, Hepatology and Nutrition (BSPGHAN) Joint Consensus Guidelines on the Diagnosis and Management of Eosinophilic Oesophagitis in Children and Adults. Gut.

[48] Kagalwalla A.F., Sentongo T.A., Ritz S., Hess T., Nelson S.P., Emerick K.M., Melin-Aldana H., Li B.U.K. (2006). Effect of Six-Food Elimination Diet on Clinical and Histologic Outcomes in Eosinophilic Esophagitis. Clin. Gastroenterol. Hepatol..

[49] Kagalwalla A.F., Shah A., Li B.U.K., Sentongo T.A., Ritz S., Manuel-Rubio M., Jacques K., Wang D., Melin-Aldana H., Nelson S.P. (2011). Identification of Specific Foods Responsible for Inflammation in Children with Eosinophilic Esophagitis Successfully Treated with Empiric Elimination Diet. J. Pediatr. Gastroenterol. Nutr..

[50] Molina-Infante J., Arias A., Barrio J., Rodríguez-Sánchez J., Sanchez-Cazalilla M., Lucendo A.J. (2014). Four-Food Group Elimination Diet for Adult Eosinophilic Esophagitis: A Prospective Multicenter Study. J. Allergy Clin. Immunol..

[51] Molina-Infante J., Arias Á., Alcedo J., Garcia-Romero R., Casabona-Frances S., Prieto-Garcia A., Modolell I., Gonzalez-Cordero P.L., Perez-Martinez I., Martin-Lorente J.L. (2018). Step-up Empiric Elimination Diet for Pediatric and Adult Eosinophilic Esophagitis: The 2-4-6 Study. J. Allergy Clin. Immunol..

[52] Kagalwalla A.F., Amsden K., Shah A., Ritz S., Manuel-Rubio M., Dunne K., Nelson S.P., Wershil B.K., Melin-Aldana H. (2012). Cow’s Milk Elimination: A Novel Dietary Approach to Treat Eosinophilic Esophagitis. J. Pediatr. Gastroenterol. Nutr..

[53] Kliewer K.L., Abonia J.P., Aceves S.S., Atkins D., Bonis P.A., Capocelli K.E., Chehade M., Collins M.H., Dellon E.S., Fei L. (2024). One-Food versus 4-Food Elimination Diet for Pediatric Eosinophilic Esophagitis: A Multisite Randomized Trial. J. Allergy Clin. Immunol..

[54] Wong J., Goodine S., Samela K., Vance K.S., Chatfield B., Wang Z., Sayej W.N. (2020). Efficacy of Dairy Free Diet and 6-Food Elimination Diet as Initial Therapy for Pediatric Eosinophilic Esophagitis: A Retrospective Single-Center Study. Pediatr. Gastroenterol. Hepatol. Nutr..

[55] Kruszewski P.G., Russo J.M., Franciosi J.P., Varni J.W., Platts-Mills T.A.E., Erwin E.A. (2016). Prospective, Comparative Effectiveness Trial of Cow’s Milk Elimination and Swallowed Fluticasone for Pediatric Eosinophilic Esophagitis. Dis. Esophagus.

[56] Wechsler J.B., Schwartz S., Arva N.C., Kim K.Y.A., Chen L., Makhija M., Amsden K., Keeley K., Mohammed S., Dellon E.S. (2022). A Single-Food Milk Elimination Diet Is Effective for Treatment of Eosinophilic Esophagitis in Children. Clin. Gastroenterol. Hepatol..

[57] Bognanni A., Fiocchi A., Arasi S., Chu D.K., Ansotegui I., Assa’ad A.H., Bahna S.L., Berni Canani R., Bozzola M., Dahdah L. (2024). World Allergy Organization (WAO) Diagnosis and Rationale for Action against Cow’s Milk Allergy (DRACMA) Guideline Update—XII—Recommendations on Milk Formula Supplements with and without Probiotics for Infants and Toddlers with CMA. World Allergy Organ. J..

[58] Leung J., Hundal N.V., Katz A.J., Shreffler W.G., Yuan Q., Butterworth C.A., Hesterberg P.E. (2013). Tolerance of Baked Milk in Patients with Cow’s Milk-Mediated Eosinophilic Esophagitis. J. Allergy Clin. Immunol..

[59] González-Cervera J., Arias Á., Navarro P., Juárez-Tosina R., Cobo-Palacios M., Olalla J.M., Angueira-Lapeña T., Lucendo A.J. (2022). Tolerance to Sterilised Cow’s Milk in Patients with Eosinophilic Oesophagitis Triggered by Milk. Aliment. Pharmacol. Ther..

